# Impact of left atrial appendage morphology on thrombus formation after successful left atrial appendage occlusion: Assessment with cardiac-computed-tomography

**DOI:** 10.1038/s41598-018-19385-z

**Published:** 2018-01-26

**Authors:** Wulf Dieker, Michael Behnes, Christian Fastner, Benjamin Sartorius, Annika Wenke, Ishar Sing-Gill, Ibrahim El-Battrawy, Jürgen Kuschyk, Theano Papavassiliu, Ursula Hoffmann, Kambis Mashayekhi, Stefan O. Schoenberg, Martin Borggrefe, Thomas Henzler, Ibrahim Akin

**Affiliations:** 1 First Department of Medicine, University Medical Centre Mannheim, Faculty of Medicine Mannheim, University of Heidelberg, Mannheim, Germany; 2 Division of Cardiology and Angiology II, University Heart Center Freiburg-Bad Krozingen, Bad Krozingen, Germany; 3 Institute of Clinical Radiology and Nuclear Medicine, University Medical Center Mannheim, Faculty of Medicine Mannheim, University of Heidelberg, Heidelberg, Germany

## Abstract

A standardized imaging algorithm by cardiac computed tomography angiography (cCTA) (LOVE-view) was used in 30 patients to evaluate the influence of different left atrial appendage (LAA) morphologies on development of thrombosis in the LAA 6 months after implantation of an occlusion device (Watchman or Amplatzer-Cardiac-Plug) in patients with non-valvular atrial fibrillation, CHA_2_DS_2_-VASc-Score >1 and a contraindication for oral anticoagulation. The distribution of different LAA morphologies was 40% windsock, 17% broccoli and 43% chicken wing type. There was no significant difference in the level of thrombosis regarding LAA morphology or the type of chosen occlusion device. The rates of complete LAA thrombosis was 40% in broccoli type, 33% in windsock and 15% in chicken wing type. Independently of LAA type, 13% had none and 60% incomplete thrombosis. The ratio of density (LA/LAA) was 0.14 in patients with complete thrombosis and 0.67 in those with none or incomplete thrombosis. cCTA and the LOVE-view-imaging-algorithm were shown to be a valuable method for standardized imaging in clinical routine in a greater set of patients. Surprisingly thrombosis of the occluded LAA was still in progress in most cases at 6 months, whereas further studies are needed defining its clinical consequences, especially for the selection of the optimal post-procedural antithrombotic treatment strategy.

## Introduction

Implantation of left atrial appendage (LAA) occlusion devices prevents  effectively and safely cardio-embolic stroke in patients with non-valvular atrial fibrillation, especially in those with contraindication for oral anticoagulation or a high risk for major bleedings^1–3^. Due to its predisposing morphology for blood stasis and endothelial dysfunction the LAA is the origin of 90% of cardiac thromboembolism^4^. However, LAA morphology is highly variable regarding size, number of lobes and the inherent configuration. Generally the LAA configuration is categorized into different types namely windsock, chicken wing, broccoli and cauliflower^5,6^. These four LAA types do not only complicate the individual percutaneous implantation of LAA occlusion devices, they are also associated with different risks of cardio-embolic stroke independently from patients’ individual CHA2DS2-VASc score^5^. For example, patients with a chicken wing configuration are less likely to have a cardio-embolic stroke when compared to patients with other LAA types^5^.

At present, the Watchman (Boston Scientific, Natrick, MA, USA) and the Amplatzer Cardiac Plug (St. Jude Medical, St Paul, MN, USA) devices are the most commonly implanted LAA occlusion devices^7^. Both of them seal the ostium of the LAA from the blood flow and are expected to be covered by endothelialization within the following months. However, because of the afore mentioned anatomic variation an incomplete sealing and consequent residual blood flow in the LAA may occur. Until now, trans-esophageal echocardiography (TEE) is the most widely used imaging modality for post implantation evaluation of LAA occlusion devices. However, TEE is often limited for the evaluation of thrombus formation and remaining blood flow within the occluded LAA^8–10^. Recently, cardiac computed tomography angiography (cCTA) has been proposed as a promising alternative to TEE for post implantation evaluation of the LAA^11^.

In contrast to TEE, cCTA allows excellent non-invasive anatomic visualization of the complete LAA including the direct visualization of LAA thrombus and LAA occlusion device placement^11^. However, no study has yet applied cCTA for the evaluation of LAA thrombosis after successful LAA closure (LAAC). Therefore, this study evaluates for the first time the incidences and degree thrombosis within the LAA 6 months after successful LAAC depending on LAA morphologies as well as the implanted occlusion devices.

## Methods

Patients with non-valvular atrial fibrillation and indication for oral anticoagulation due to a CHA_2_DS_2_-VASc score ≥2 were included in this single-center, prospective, observational non-randomized study since June 2014. Inclusion criteria were age ≥18, a relative or absolute contraindication for oral anticoagulation, which was major or recurrent bleeding, HAS-BLED score ≥3 or intolerance to oral anticoagulation in most cases. Exclusion criteria were single episode of atrial fibrillation or a treatable cause, planned catheter ablation of atrial fibrillation or electrical cardioversion within 30 days prior or after LAAC, congestive heart failure at NYHA stage IV, myocardial infarction within the last 3 months, atrial septum defect or interventional or surgical occlusion of ASD, mechanical heart valve, status after heart transplant, symptomatic carotid stenosis, transient ischemic attack or stroke within last 30 days, intracerebral bleeding within the last 3 months, acute infection, existing or planned pregnancy, and existing cardiac thrombus. Informed consent was obtained from all patients. The study was carried out according to the principles of the declaration of Helsinki and was approved by the medical ethics committee II of the Faculty of Medicine Mannheim, University of Heidelberg, Germany. The LAA occlusion device implantation was performed by experienced interventional cardiologists and the selection of one of the two devices (Watchman or Amplatzer Cardiac Plug) was based on individual anatomic considerations. The detailed procedure and post-procedural measures have been previously described by our working group^12^. After LAAC 100 mg/d of acetylsalicylic acid was prescribed permanently and 75 mg/d clopidogrel for at least 6 months with a loading dose of 250 mg, respectively 600 mg if not taken before.

### Cardiac computed tomography angiography imaging at 6 months of follow-up

cCTA was performed 6 months after successful LAAC. All cCTA scans were performed using a 2 × 192-slice 3^rd^ generation dual-source CT (Siemens Force, Siemens Healthineers, Forchheim, Germany) using a dual-energy scan mode. Acquisition parameters for the dual energy cCT were tube voltage 90 kV (tube A), 150 kV with tin filter (tube B) with topogram dependent tube current modulation for both tubes; detector collimation 2 × 192 × 0.6 mm; slice thickness 0.6 mm, increment 0.5 mm. All cCTA acquisitions were performed with retrospective ECG-gating and bolus triggering technique with a region of interest (ROI) placed in the descending aorta and 100 HU threshold. For i.v. contrast 80 cc of iodinated contrast material (Imeron 400, Bracco, Milan, Italy) were administered via a 18 G cubital catheter with an injection rate of 5 ml/sec followed by a 50 ml saline flush.

A systematic approach to evaluate implanted LAA occlusion devices has been recently described by the so called “LOVE” views, revealing optimal device-related angulation allowing optimal evaluation of the device post implantation^11^. In addition, one cardiologist and cardiac radiologist measured the contrast attenuation within the LAA behind the device as well as in the LA.

### Statistical analysis and data Availability

Statistical analyses were performed with IBM® SPSS® Statistics Version 21.0.0.0 (IBM, Armonk, NY). Descriptive statistics are given as medians (25^th^ and 75^th^ percentiles), mean (STD) or as total numbers with group-related percentages. Fisher’s exact test was used in the analysis of contingency tables. The datasets generated during the current study are available from the corresponding author on reasonable request. Univariate ANOVA was used to analysis differences between the mean densities between different stages of thrombosis. Due to homogeneity of variance, we used Welch’s test and Games-Howell post-hoc test.

## Results

### Baseline characteristics and distribution of LAA morphologies

cCT images of a total of 30 patients (mean age 75 years; 76.7% male) were collected and evaluated by using the “LOVE view” imaging algorithm at 6 months after successful LAAC. The demographic and clinical baseline characteristics are shown in Table 1. The prevalence of different LAA morphologies was 40% (n = 12) windsock, 16.7% (n = 5) broccoli and 43.3% (n = 13) chicken wing type, respectively. No cauliflower type was detected. In the present study cohort, no stroke or transient ischaemic attack (TIA) did occur during follow-up.

**Table 1:** Demographic and clinical baseline characteristics of the study cohort.

Characteristic	Value
Patients, n (%)	30
Male, n (%)	23 (77)
Age, y	78.5 (73.25–82)
Height, cm	172 (168.5–176.75)
Weight, kg	82.5 (71.38–90)
BMI, kg/m^2^	25.9 (24.5–30.2)
Hypertension, n (%)	28 (93)
Diabetes mellitus, n (%)	9 (30)
Stroke, n (%)	4 (13)
TIA, n (%)	2 (7)
ICB, n (%)	5 (17)
Coronary artery disease, n (%)	17/57)
Peripheral vascular disease, n (%)	2 (7)
Renal failure, n (%)	11 (37)
Liver Disease, n (%)	3 (10)
AF type n (%)	
Paroxysmal	15 (50)
Persistent	6 (20)
Permanent	9 (30)
Labile INR, n (%)	2 (7)
CHA_2_DS_2_-VASc score	4 (3–5)
HAS-BLED score	4 (3–4.75)
Prior bleeding, n (%)	25 (83)
GI	17 (56.7)
ICB	5 (16.7)
Urinary	2 (6.7)
Others	7 (23.4)

Values are given as medians (1. and 3. quartile) or as total number (percentage). Abbreviations: GI: gastrointestinal; AF: atrial fibrillation; OAC: oral anticoagulation, INR: international normalized ratio; ICB: intracranial bleeding; TIA: transient ischemic attack.

### Different stages of LAA thrombosis after percutaneous LAA occlusion

**Figure 1:**
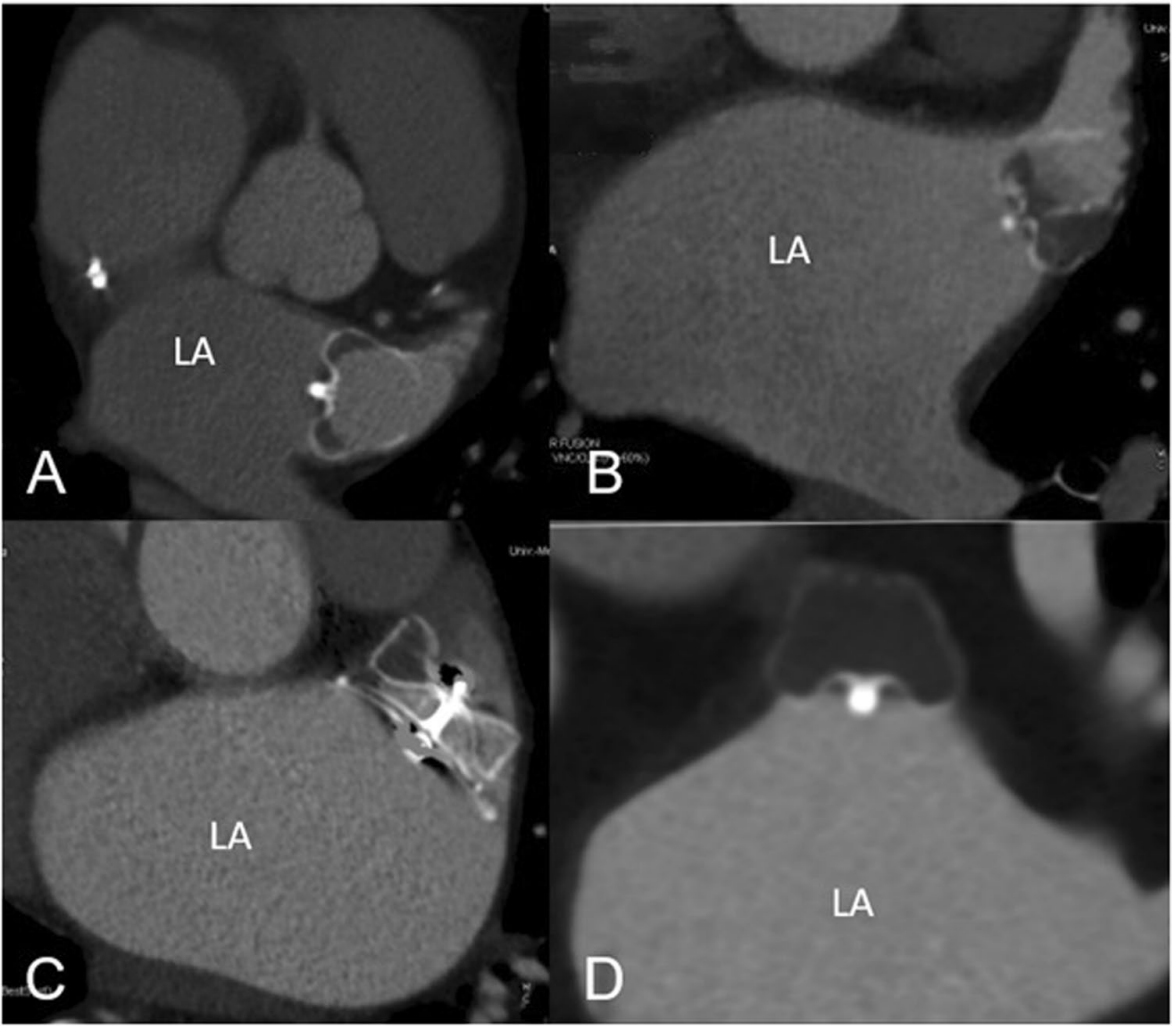
Illustrating different stages of LAA thrombosis using dual-energy cardiac CT. Panel A shows a hyperdense contrast agent flow in all parts of the LAA. Panel B shows a beginning thrombosis of the occlusion device with isodense flow, while in Panel C thrombosis has expanded to parts of the LAA with hypodense contrast agent flow in the remaining LAA. Panel D is an example of good device and LAA thrombosis with complete coverage of all lobes.

Figure 1 shows exemplary different stages of observed LAA thrombosis from none (A), beginning thrombosis limited to parts of the occlusion device (B) or the LAA (C) to complete thrombosis of all parts of the LAA (D). Only the last finding was rated as complete thrombosis.

**Figure 2:**
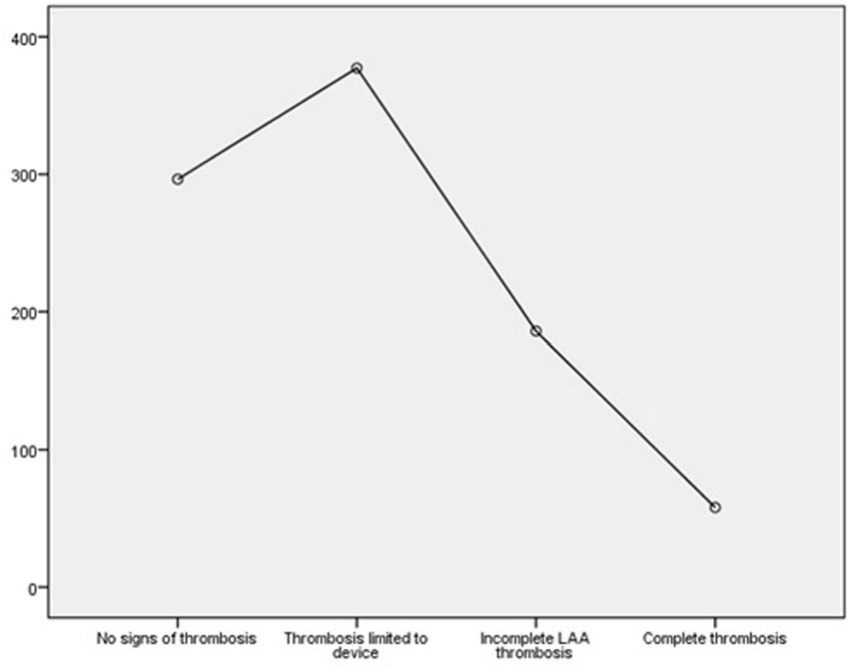
Mean density (HU) in different levels of thrombosis (y-axis). Mean density was: no signs of thrombosis: 296.43 (STD 197,8); device limited thrombosis: 377.30 (STD 112.3); incomplete LAA thrombosis: 185,98 (STD 161.61); complete thrombosis: 57.94 (STD 37.33). Post-hoc test (Games-Howell) revealed a significant difference between device limited thrombosis compared to incomplete LAA thrombosis (191.33, 95%-CI [6.18 –, 376.47]; p = 0.042) and complete thrombosis (319.36; 95%-CI [173.00 –, 465.72]; p = 0.001).

The mean density in all left atria was 401.35 (STD 174.8) Hounsfield Units (HU) (measured 2 cm before the device), and significantly differing  between LAAs with complete thrombosis (57.94 (STD 37.32)) and those with none or incomplete thrombosis (266.94 (STD 167.2) HU) (p value 0.004). Behind the devices with remaining contrast agent flow the mean density was 308.17 (STD 177.4) HU, in those with thrombi located inside the device (as in B, Fig. 1) it was 377.03 (STD 112.27) HU. The ratio of density between LA and LAA was 0.14 at patients with completed thrombosis and 0.67 in those with none or incomplete thrombosis. An ANOVA comparison of the mean density (HU) in all left atrial appendages was conducted to evaluate the null hypothesis, that there is no difference between the different grades of thrombosis. The independent variable, level of thrombosis, included four groups: no signs of thrombosis, thrombosis limited to the device, incomplete LAA thrombosis and complete thrombosis of LAA. The mean density differed statistically significant for the different levels of thrombosis, Welchs’s, F(3, 9.060) = 17.042, p < 0.001. Games-Howell post-hoc analysis revealed a significant difference between device limited thrombosis compared to incomplete LAA thrombosis (191.33, 95%-CI [6.18–376.47]; p = 0.042) and complete thrombosis (319.36; 95%-CI [173.00–465.72]; p = 0.001) (Fig. 2).

### LAA thrombosis related to LAA morphology after percutaneous LAA occlusion

The broccoli type had the highest rate of complete LAA thrombosis at 6 months (40%), followed by the windsock type (33%), wheras only 15% of the chicken wing formed LAAs had completed thrombosis at 6 months (Table 2). However, this difference was not statistically significant (Fishers’s exact test p-value = 0.484). Overall, independently of LAA types, 73% of all patients had none (13%) or incomplete thrombosis (60%) at 6 months.

**Table 2:** Level of LAA thrombosis depending on different LAA morphologies.

LAA type	Complete thrombosis	Incomplete thrombosis of LAA	Thrombosis limited to device	No thrombosis	P-value
Windsock	4 (33)	4 (33)	2 (17)	2 (17)	0.484
Broccoli	2 (40)	1 (20)	2 (40)	0 (0)	
Chicken Wing	2 (15)	6 (46)	3 (23)	2 (15)	

Values are total numbers (percentage). Analysis of contingency tables by Fishers’s exact test p-value = 0.484 revealed no significant difference.

### LAA thrombosis related to the implanted LAA occlusion device

In 60% the implanted occlusion device was a Watchman, in 40% an Amplatzer Cardiac Plug. Regarding the device type, 22% of all patients with an implanted Watchman device had complete thrombosis compared to 33% of the Amplatzer Cardiac Plug patients (Table 3), but the difference had no statistical significance (Fishers’s exact test p-value = 0.396).

**Table 3:** Level of LAA thrombosis depending on device type.

Device type	Complete thrombosis	Incomplete thrombosis of LAA	Thrombosis limited to device	No thrombosis	P-value
Watchman	4 (22)	5 (28)	6 (33)	3 (17)	0.396
Amplatzer	4 (33)	6 (50)	1 (8)	1 (8)	

Values are total numbers (percentage). Analysis of contingency tables by Fishers’s exact test p-value = 0.396 revealed no significant difference.

## Discussion

The present observational study analyzes for the first time the incidences of LAA thrombosis 6 months after successful percutaneous implantation of left atrial appendage occlusion devices in relation to different LAA morphologies and implanted occlusion devices being assessed by cardiac computed tomography imaging.

It was demonstrated that complete LAA thrombosis after successful device implantation occurs with mean overall incidence of 27%. Numerical differences were found in between different LAA morphologies. Noteworthy, a higher rate of complete thrombosis in the broccoli and windsock type was found (40% and 33% respectively) compared to the chicken wing type (15%), which was also the one with the lowest risk for cardio-embolic stroke in non-occluded patients^5^.

Incomplete thrombosis was seen in most patients (73%), including a minor part of patients without any signs of LAA thrombosis (13%). The chosen type of device (Watchman vs. Amplatzer Cardiac Plug) had no influence on the level of thrombosis.

The presented novel findings on incidences of LAA thrombosis are of descriptive and observational character. Therefore, this study delivers relevant scientific evidence about the mid-term follow-up of implanted LAA occlusion devices and reveals insights about LAA thrombosis, which has never been demonstrated before, except for this observational study.

However, currently it is not known if missing or incomplete thrombosis has an influence on the assured protection against cardio-embolic stroke from the LAA by the device. In this cohort, no stroke or TIA did occur during follow up. This study showed for the first time, that the process of thrombogenesis lasts longer than assumed^13^. Whether there are patients in which the LAA is not occluded at all should be the subject of further cCTA investigation at long term follow-up. As a main result, due to these new insights of LAA thrombogenesis, we recommend further studies defining the clinical consequences of incomplete thrombosis after LAA occlusion. Furthermore, the present results may potentially influence ongoing discussion about optimal post-procedural antithrombotic drug therapy and duration^14,15^. If relevant and along with clinical studies in this population a different antiplatelet or anticoagulation strategy may be needed to be discussed.

The left atrial appendage represents the origin of 90% of cardiac thromboembolism leading to severe ischemic stroke, which is the most common reason for disability in adults and is associated with a high burden of morbidity and mortality^4^. In about 25% of all ischemic strokes the heart, usually atrial fibrillation, is the causative organ^16^. In the last years the interventional approach by implementation of occlusion devices in the LAA is becoming a more and more useful supplement in treatment options to the well-established oral anticoagulation in selected patients^1^. In this procedure, the anatomic variations of the LAA do not only influence the individual implantation, they are also an independent risk factor for cardio-embolic stroke^5^. However TEE, which is the standard method for post implementation device evaluation, is limited especially in assessment of LAA thrombosis and remaining blood flow behind the occlusion device. This study shows that cCTA is well suited for post-procedural assessment and allows good estimation of the degree of thrombosis and remaining blood flow inside the implanted device and the remaining LAA. Besides acceptable amount of radiation and contrast agent in modern cCTA imaging^17^, the main advantage over TEE consists in the superior assessment by sharp image acquisition of cardiac structures and blood flow within or behind the LAA occlusion device.

## Study Limitations

The present study represents an observational study of a small subset of patients undergoing cCTA after successful LAAC at mid-term follow-up. Despite the novelty of the presented findings of LAA thrombosis, the present study lacks several limitations of such small-scaled studies. Due to its descriptive and observational character, clinical implications cannot be withdrawn from the presented data. The outlined clinical implications in the discussion section may only be speculated. The evaluation of the level of thrombosis within the LAA was done by visual assessment. No statement can be made whether there is a correlation between different stages of thrombosis and reduced effectiveness of the stroke-prevention by the LAA occlusion device. Assessment of incomplete coverage of lobes, peri-device leaks and indirect signs of neo-endothelialization was beyond the scope of the present analysis and will be analyzed in more detail elsewhere.

## Conclusions

cCTA is a noninvasive and precise procedure for post-procedural assessment of LAA occlusion devices and an alternative to TEE, especially in the assessment of the LAA behind the device. There was no significant difference in level of thrombosis regarding different types of LAA-morphology or different chosen devices. We assume that the main confounding factors are an incomplete coverage of lobes and/or peri-device leaks, that lead to a disturbance of LAA thrombosis by persistent blood flow. The novel finding of this study is, that 6 months after LAAC the intended thrombosis of the LAA is still in progress in most cases. Therefore, further studies are necessary to find out if incomplete thrombosis within the LAA has clinical consequences. If relevant these could be a noteworthy factor in the debate about the optimal post-procedural antithrombotic strategy or drug selection.

## References

[1] Camm AJ (2012). 2012 focused update of the ESC Guidelines for the management of atrial fibrillation: an update of the 2010 ESC Guidelines for the management of atrial fibrillation. Developed with the special contribution of the European Heart Rhythm Association. European heart journal.

[2] Holmes DR (2009). Percutaneous closure of the left atrial appendage versus warfarin therapy for prevention of stroke in patients with atrial fibrillation: a randomised non-inferiority trial. Lancet.

[3] Park JW (2011). Left atrial appendage closure with Amplatzer cardiac plug in atrial fibrillation: initial European experience. Catheterization and cardiovascular interventions: official journal of the Society for Cardiac Angiography & Interventions.

[4] Blackshear JL, Odell JA (1996). Appendage obliteration to reduce stroke in cardiac surgical patients with atrial fibrillation. The Annals of thoracic surgery.

[5] Di Biase L (2012). Does the left atrial appendage morphology correlate with the risk of stroke in patients with atrial fibrillation? Results from a multicenter study. Journal of the American College of Cardiology.

[6] Regazzoli D (2015). Left Atrial Appendage: Physiology, Pathology, and Role as a Therapeutic Target. BioMed research international.

[7] Saw J, Lempereur M (2014). Percutaneous left atrial appendage closure: procedural techniques and outcomes. JACC. Cardiovascular interventions.

[8] Onalan O, Crystal E (2007). Left atrial appendage exclusion for stroke prevention in patients with nonrheumatic atrial fibrillation. Stroke.

[9] Viles-Gonzalez JF (2012). The clinical impact of incomplete left atrial appendage closure with the Watchman Device in patients with atrial fibrillation: a PROTECT AF (Percutaneous Closure of the Left Atrial Appendage Versus Warfarin Therapy for Prevention of Stroke in Patients With Atrial Fibrillation) substudy. Journal of the American College of Cardiology.

[10] Viles-Gonzalez JF (2012). Incomplete occlusion of the left atrial appendage with the percutaneous left atrial appendage transcatheter occlusion device is not associated with increased risk of stroke. Journal of interventional cardiac electrophysiology: an international journal of arrhythmias and pacing.

[11] Behnes M (2016). –LAA Occluder View for post-implantation Evaluation (LOVE)–standardized imaging proposal evaluating implanted left atrial appendage occlusion devices by cardiac computed tomography. BMC medical imaging.

[12] Fastner C (2016). Left atrial appendage morphology, echocardiographic characterization, procedural data and in-hospital outcome of patients receiving left atrial appendage occlusion device implantation: a prospective observational study. BMC cardiovascular disorders.

[13] Meier B (2015). EHRA/EAPCI expert consensus statement on catheter-based left atrial appendage occlusion. EuroIntervention: journal of EuroPCR in collaboration with the Working Group on Interventional Cardiology of the European Society of Cardiology.

[14] Tzikas A, Bergmann MW (2016). Left atrial appendage closure: patient, device and post-procedure drug selection. EuroIntervention: journal of EuroPCR in collaboration with the Working Group on Interventional Cardiology of the European Society of Cardiology.

[15] Jalal Z (2017). Percutaneous left atrial appendage closure followed by single antiplatelet therapy: Short- and mid-term outcomes. Archives of cardiovascular diseases.

[16] Marini C (2005). Contribution of atrial fibrillation to incidence and outcome of ischemic stroke: results from a population-based study. Stroke.

[17] Henzler T, Fink C, Schoenberg SO, Schoepf UJ (2012). Dual-energy CT: radiation dose aspects. AJR. American journal of roentgenology.

